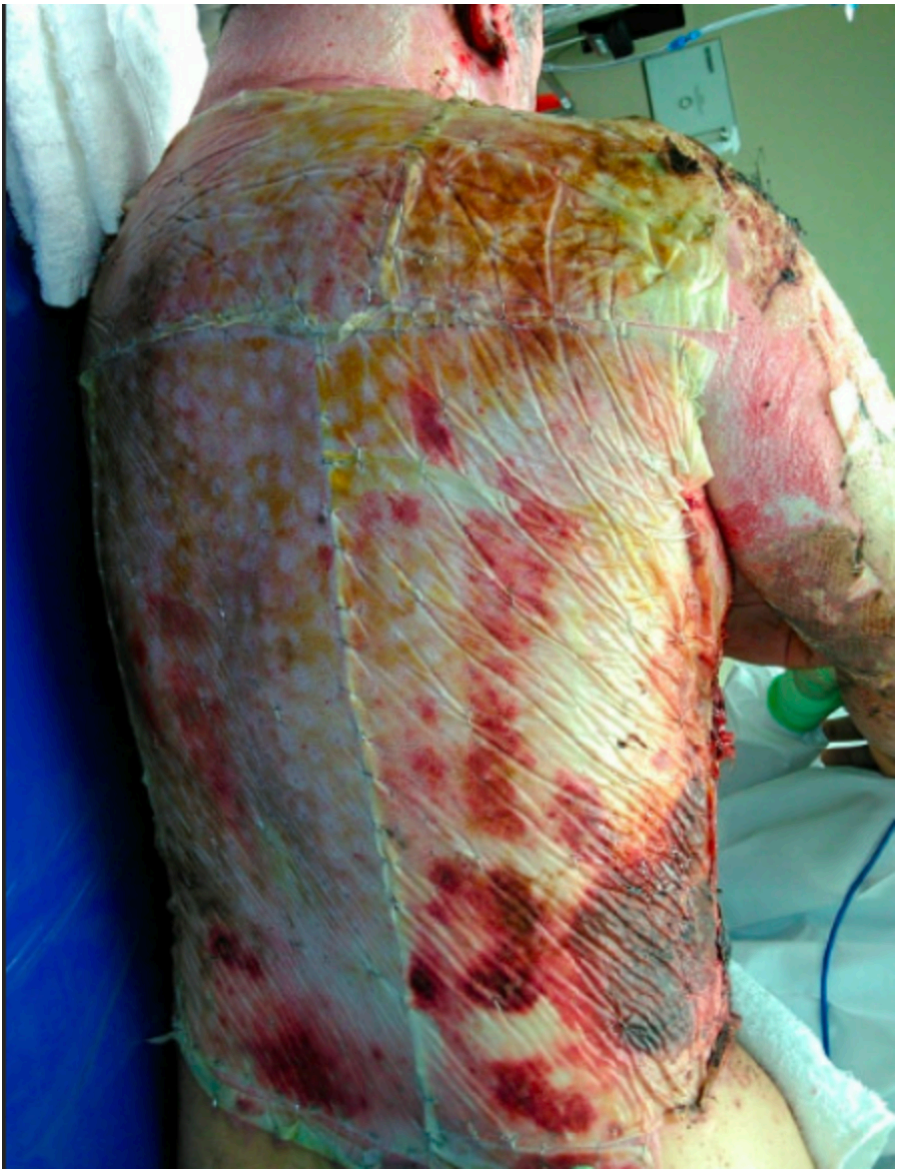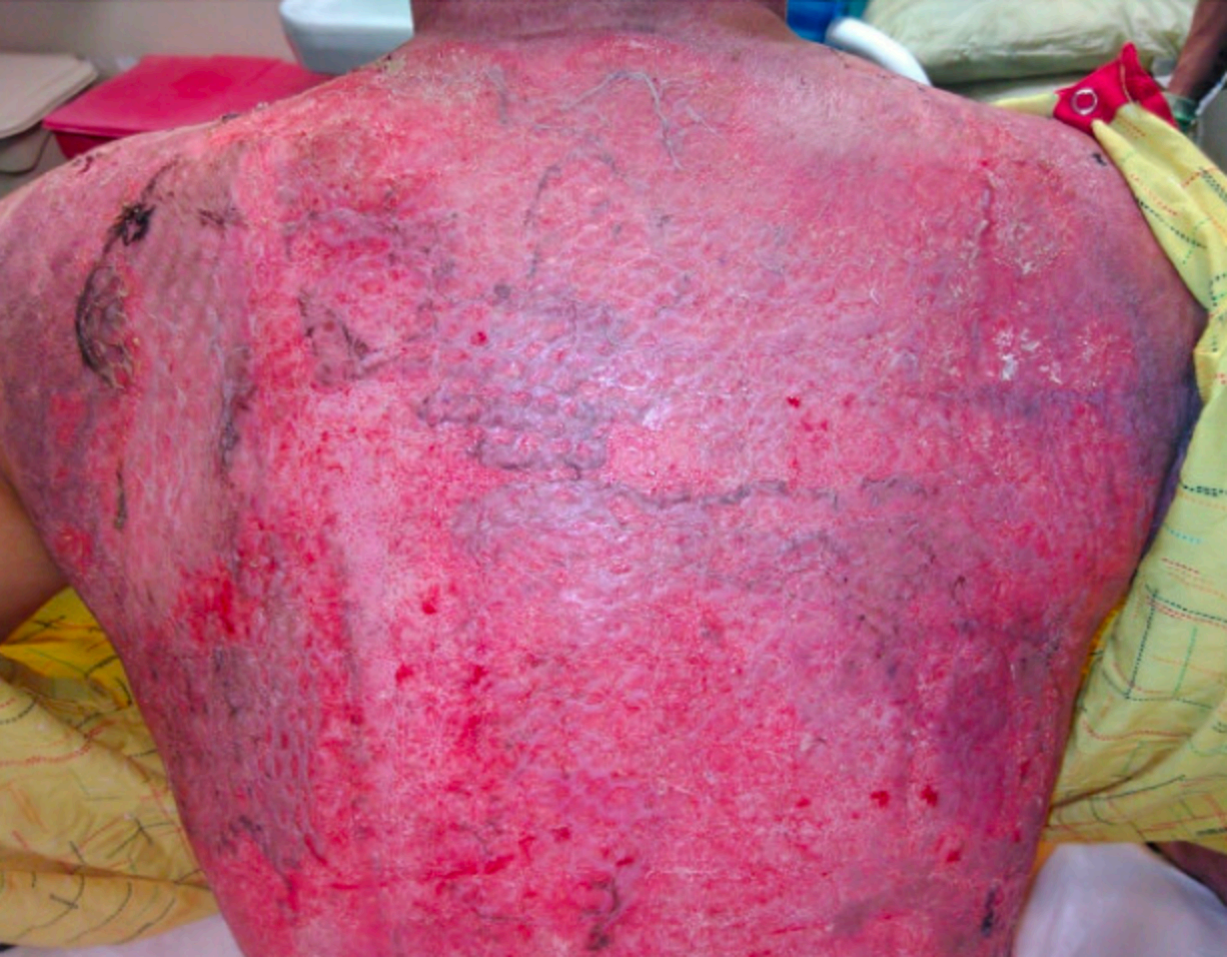# 861 Treatment of Full-Thickness Burns with Biodegradable Polyurethane Matrix and Autologous Skin Cell Suspension: Case Series

**DOI:** 10.1093/jbcr/iraf019.392

**Published:** 2025-04-01

**Authors:** Jaina Eckert, Hannah Chaudhury, Liza Garcia, Alan Pang, John Griswold

**Affiliations:** Texas Tech University Health Sciences Center School of Medicine; Texas Tech University Health Sciences Center School of Medicine; Texas Tech University Health Sciences Center School of Medicine; Texas Tech University Health Sciences Center School of Medicine; Texas Tech University Health Sciences Center School of Medicine

## Abstract

**Introduction:**

Over the past decade, novel options have provided promising approaches into the conundrum of how to best treat full thickness burns. The primary objective of proper resuscitation and adequate skin coverage is a balancing act between fighting infections, insensible loss, and metabolic imbalances. Previously, dermal substitutes have been the traditional treatment option for full thickness burns, but availability and acquisition delays treatment, increasing the risk of mortality despite advances in treatment options. Recent studies have shown the use of a dermal substitute as an initial treatment option, followed by the use of an autologous skin cell suspension with or without skin graft treatment for severely burn patients. This case series describes the treatment course of severely burn patients using a biodegradable polyurethane matrix (BPM) as the dermal substitute with simultaneous application of an autologous cell harvesting device (ACH) as a unique approach for initial treatment of full thickness burns.

**Methods:**

A retrospective chart review using EHR to obtain deidentified data was done to isolate burn patients within the past January 1,2020-December 31,2022 who were treated simultaneously with BPM as dermal substitute with simultaneous application of ACH. Data was collected from 4 patient charts who met inclusion criteria and underwent the treatment. The primary endpoint was assessing % epithelialization that occurred with the simultaneous application, and secondary endpoints included incidence of infection/septic episodes, ICU length of stay, adverse events and mortality.

**Results:**

Our case series focuses on 4 male patients with TBSA ranges of 30-90% who showed early epithelization upon delamination of BPM. Case 1 had 3 comorbidities, including obesity, smoking and alcohol use. Case 2 had one documented comorbidity, obesity. Case 3 had zero documented comorbidities. Case 4 had a history of IV drug use. Despite varying comorbidities, there was no obvious association to % epithelization outcomes. All four patients successfully underwent BPM delamination without adverse events or site infections. Although only a small fraction of each patient’s TBSA received the simultaneous treatment of BPM and ACH, the areas that underwent this approach resulted in as much as 50% epithelization of the treated burn wound.

**Conclusions:**

This demonstrates clinical significance which merits further investigation to determine statistical significance. This simultaneous application of BPM with ACH presents an opportunity to minimize delay in treatment and improve wound healing by reducing fluid loss, metabolic imbalances, and potential risk for infection.

**Applicability of Research to Practice:**

This technique can be considered an option for patients with full thickness injuries adjacent to indeterminate thickness injuries with potential intact dermal elements to minimize delay in re-epithelialization.

**Funding for the Study:**

N/A